# Spontaneous rupture and upper gastrointestinal bleeding of solid pseudopapillary neoplasm of the pancreas

**DOI:** 10.1093/jscr/rjac462

**Published:** 2022-10-10

**Authors:** Fernando Revoredo, Fritz Kometter, Juan Núñez, Julio León, Juan Luna, Jorge Valdes

**Affiliations:** Department of General Surgery, Clínica Internacional, Lima, Perú; Department of General Surgery, Clínica Internacional, Lima, Perú; Department of General Surgery, Clínica Internacional, Lima, Perú; Department of Gastroenterology, Clínica Internacional, Lima, Perú; Department of General Surgery, Clínica Internacional, Lima, Perú; Division of Pathology, Clínica Internacional - Instituto de Patología y Biología Molecular Arias Stella, Lima, Perú

## Abstract

The solid pseudopapillary neoplasm (SPN) of the pancreas is an uncommon, low-grade malignant tumour, mostly seen in young women. We report a rare case of a 44-year-old female who presented with spontaneous rupture and upper gastrointestinal bleeding. The emergency endoscopy revealed a 2 cm bleeding ulcer on the duodenal bulb. A computer tomography scan (CT scan) showed a 6.7 cm mass, with solid and cystic components arising in the head of the pancreas. After achieving haemostasis, she was discharged. Two months later, a new CT scan showed a persistent 6 cm mass in the head of the pancreas, now containing air and communicating with the duodenal lumen. The patient was successfully treated by pancreatoduodenectomy. Histopathological examination showed a T3N0M0 SPN with immunohistochemical expression of β-catenin, synaptophysin, vimentin and progesterone receptor, and negativity for chromogranin. The labelling index of Ki 67 was 2%. No recurrence was present after 2 years of follow-up.

## INTRODUCTION

The solid pseudopapillary neoplasm (SPN) of the pancreas is a low-grade malignant tumour that is composed of poorly cohesive epithelial cells, forming solid and pseudopapillary structures [1, 2]. The specific line of pancreatic epithelial differentiation of this neoplasm is still unclear [2], but some features strongly support the theory that derives from pluripotent stem cells of the genital ridges that became translocated to the pancreas during embryogenesis [1, 2]. This hypothesis is supported by the fact that the neoplasm identical to pancreatic SPN has been described in ovaries and testis [1].

SPN is a rare entity, accounting for around 5% of all cystic neoplasms of the pancreas [1]. The vast majority of SPN (≥ 80%) occur in young women and are asymptomatic [3]. The mean age at the diagnosis is 28 years (range 7–79 years) [2]. When symptomatic, SPN may present nonspecific symptoms, such that there could be abdominal discomfort, nausea, vomiting, pain or jaundice [1]. Occasionally, SPN was discovered by rupture, haemoperitoneum and acute abdomen [2, 4].

### Cross-sectional imaging studies

Magnetic resonance imaging or computer tomography (CT) scan shows a well-demarcated mass [1] with solid and cystic components surrounded by a well-defined capsule [3], sometimes with calcifications [1]. The use of endoscopic ultrasonography-guided, fine-needle aspiration increases diagnostic accuracy [3]. The known tumour markers are not useful in the diagnostic workup because their levels are within normal values [1, 2].

Synchronous metastasis (mostly hepatic, but rarely peritoneal or in lymph nodes) is found in 10–15% of cases, whereas recurrence was observed in up to 14% of patients in a long-term follow-up [3]. SPN is associated with an excellent long-term prognosis even in a metastatic disease, with a 10-year disease-specific survival rate of 96% [2].

We report a rare case of a spontaneous ruptured SPN with upper gastrointestinal bleeding.

## CASE REPORT

**Figure 1:**
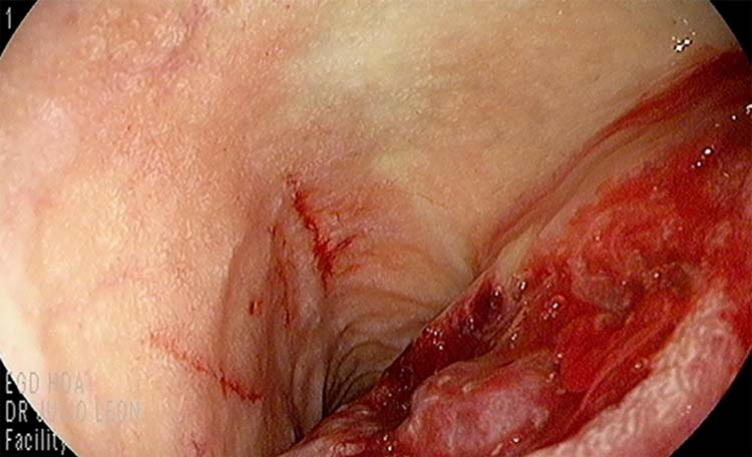
Duodenal bulb ulcer with jagged edges and an adherent clot.

**Figure 2:**
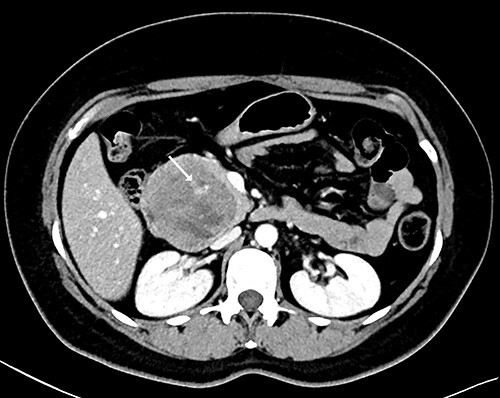
CT scan showing a well-defined heterogeneous 6.7 cm mass with solid and cystic components arising from the head of the pancreas with enhancement of contrast in the solid component, and some areas with active bleeding (white arrow). The tumour was in direct proximity to the duodenum.

Our patient is a 44-year-old healthy female who presented to the emergency department with a 1-week history of epigastric pain associated with melena, fatigue and dizziness. The abdomen was soft without tenderness, and the laboratory test showed haemoglobin at 7.3 g/dL. After two units of red blood cell transfusion, she underwent an emergency endoscopy. A 2 cm bleeding ulcer was found in the duodenal bulb with jagged edges and an adherent clot (Fig. 1). Epinephrine injection and argon plasma coagulation were used to achieve haemostasis. A CT scan revealed a well-defined heterogeneous 6.7 cm mass with solid and cystic components arising from the head of the pancreas and the enhancement of contrast in the solid component (Fig. 2). No dilatation of the common bile duct or pancreatic duct was noted. The patient was discharged without signs of bleeding and haemoglobin at 10.4 g/dL. Biopsy samples of the duodenal ulcer showed chronic inflammation, and some glandular structures suspicious of neoplasia.

**Figure 3:**
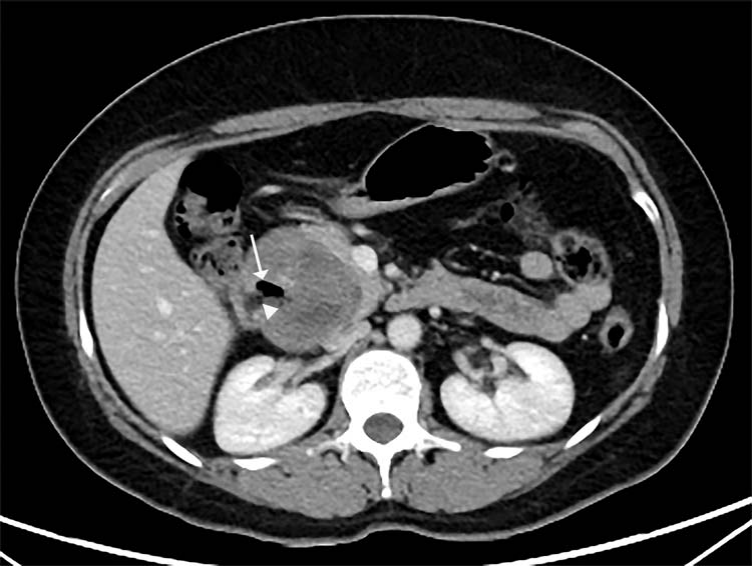
CT scan showing that tumour reduced to 6 cm, containing an air cavity (white arrow) and communicating with the duodenal lumen (arrowhead).

**Figure 4:**
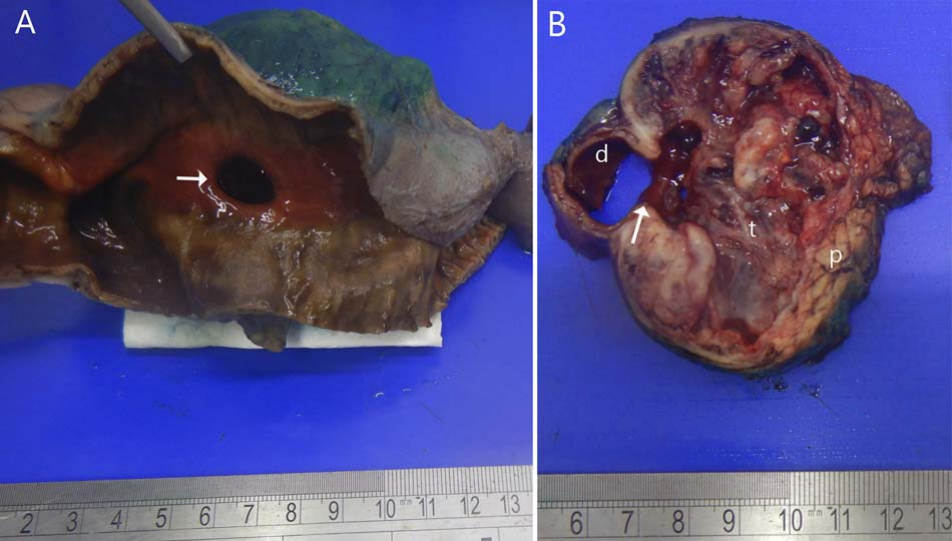
Resected specimen. (**A**) Shows the duodenal bulb ulcer (white arrow). (**B**) The gross appearance of the cut surface shows a round tumour (t) composed of solid areas, necrosis, haemorrhage and in communication with the duodenal lumen (d). Normal pancreas (p).

**Figure 5:**
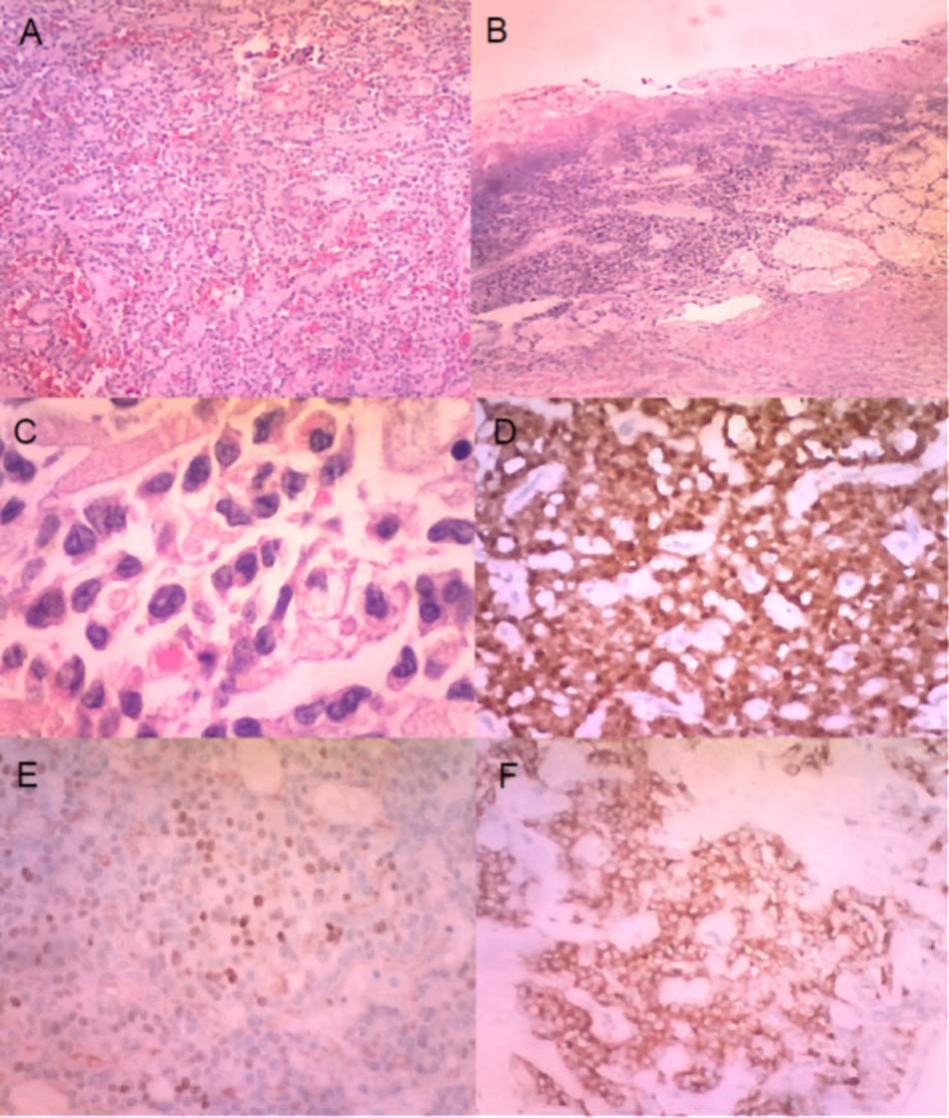
(**A**) Histologically, the tumour had neoplastic epithelioid cells growing with pseudopapillary architecture and hyalin/myxoid stroma (haematoxylin–eosin stain, 10×). (**B**) Infiltration and ulceration of the duodenum were observed (haematoxylin–eosin stain, ×10). (**C**) Some neoplastic cells contained hyaline globules (haematoxylin–eosin stain, ×40). Immunohistochemical expression of β-catenin (**D**), progesterone receptor (**E**) and synaptophysin (**F**) (×20).

Two months later, she was scheduled for surgical treatment because of the SARS CoV2 pandemic peak. A new CT scan revealed a persistent 6 cm mass in the head of the pancreas, now containing air and communicating with the duodenal lumen (Fig. 3). A slight decrease in volume was also noted. The patient underwent a pancreatoduodenectomy with standard lymphadenectomy. The procedure took 240 min and involved 200 mL of blood loss. The patient was discharged 8 days postoperatively without complications. The histopathological examination showed a neoplasm of the head of the pancreas, with a solid and cyst appearance, and abundant haemorrhagic content. The neoplasm extended to the duodenum, causing fistulation to the lumen (Fig. 4). The immunohistochemical study showed expression of β-catenin, synaptophysin, vimentin and progesterone receptor (15%) and negativity for chromogranin, E-cadherin and CD117 (Fig. 5). The labelling index of Ki 67 was 2%, and the final diagnosis was a T3N0M0 SPN. The patient did not receive postoperative chemotherapy and has remained symptom-free with no detectable recurrence for 2 years of follow-up.

## DISCUSSION

The incidence of ruptured SPN was reported at 2.7%, most of which was after abdominal trauma [4]. Spontaneous rupture is a quite rare complication reported in <1% of all SPN [4]. We found at least 16 cases [4–18], including the present case, of spontaneous ruptured SPN (Table 1), all in female patients, with mean age of 22 years (6 years less than general presentation), and mean size of 11 cm.

**Table 1:** Reported cases of SPN with spontaneous rupture

**No.**	**Author**	**Year**	**Gender**	**Age (yr)**	**Clinical presentation**	**Neoplasm location in pancreas**	**Size (cm)**	**Surgical procedure**	**Follow-up (yr)**	**Recurrence**
1	Bombí [5]	1984	Female	22	Pain Haemoperitoneum	Body	12	DP	2	No
2	Todani [6]	1988	Female	16	Pain Haemoperitoneum	Tail	9	DP	5	No
3	Hernandez [7]	1989	Female	22	Pain Haemoperitoneum	Tail	16	DP	1	No
4	Jeng [8]	1993	Female	26	Pain Haemoperitoneum	Body	13	DP	5.5	No
5	Panieri [9]	1998	Female	34	Pain Haemoperitoneum	Body	12	DP	Dead	
6	Omori [10]	2005	Female	31	Pain Haemoperitoneum	Body	10	DP	3	No
7	Kyokane [11]	2008	Female	51	Pain Haemoperitoneum	Body/tail	11	DP	8	Yes (6 yr 6mo)
8	Takamatsu [4]	2013	Female	13	Pain Haemoperitoneum	Tail	5	EN	2	No
9	Huang [12]	2013	Female	29	Pain Haemoperitoneum	Body	17	DP	1	No
10	Pattanshetti [13]	2014	Female	12	Pain Haemoperitoneum	Body	13	DP	NA	NA
11	Rampersad [14]	2018	Female	8	Pain Haemoperitoneum	Tail	7	DP	3	No
12	Natsume [15]	2018	Female	22	Pain	Head	8	PD	2	No
13	Nambada [16]	2019	Female	13	Pain Haemoperitoneum	Tail	NA	DP	1.5	No
14	Xu [17]	2019	Female	22	Pain Haemoperitoneum	Body	8	DP	1	No
15	da Silva [18]	2021	Female	31	Pain Melena	Head	12	PD	1.5	No
16	Current	2022	Female	44	Pain Melena	Head	6	PD	2	No

DP: distal pancreatectomy, EN: enucleation, PD: pancreatoduodenectomy, NA: Not available

Little is known about the pathogenesis of spontaneous rupture [15]. It has been hypothesized that it results from the infiltration of the neoplasm capsule and abrupt massive haemorrhage with the increased pressure inside the neoplasm. SPN had a natural tendency to haemorrhage inside the tumour, and the cystic part of it results from the degeneration following intramural haemorrhage [4, 15]. All spontaneous ruptured SPN located in the distal pancreas presented with haemoperitoneum and two of the three spontaneous ruptured SPN located in the head of the pancreas presented upper gastrointestinal bleeding. It might be explained by the fact that, although the neoplasm is grossly well-demarcated by a fibrous capsule, microscopically it focally infiltrates the capsule, surrounding the pancreatic tissue or duodenum [1], thereby weakening this structure, and giving rise to rupture risk mainly when an abrupt increase of intra-neoplastic pressure (massive haemorrhage) or external trauma happens.

Surgery, including the resection of distant metastases, is the treatment of choice, with an excellent long-term prognosis even when metastatic [2]. As this neoplasm is considered a low-grade malignant tumour, an oncological surgical approach with standard lymphadenectomy should be the treatment of choice.

The positive nuclear and cytoplasmatic staging for β-catenin is essential for the histopathologic diagnosis [1]. In addition, SPN is also positive for CD10, progesterone receptor, vimentin, cyclin D1, synaptophysin, CD56, CD117 and aberrant expression of E-cadherin. The differential diagnosis includes acinar cell markers (trypsin, chymotrypsin and BCL10) or neuroendocrine markers (chromogranin and pancreatic hormones) [1, 2].

Several attempts have been made to identify factors associated with SPN recurrence (gender, age, neoplasm size, positive surgical margins, distant metastases, perineural invasion, angioinvasion, deep infiltration of surrounding tissues and Ki-67 index), but the results were not conclusive and sometimes contradictory [2]. Some authors asserted that the neoplasm rupture, even during surgery, can potentially lead to neoplastic cell implantation in the peritoneal cavity [16] and might be a risk factor for recurrence [3, 15]. On the other hand, the recurrence has been reported in only one patient (6%) with the spontaneous ruptured SPN, after 6 years and 6 months of follow-up [11]. The short periods of follow-up (< 5 years) studies reporting spontaneous ruptured SPN could be the reason for this low recurrence.

Finally, there are limited data concerning the role of neoadjuvant and adjuvant chemotherapy and radiotherapy [3].

## CONCLUSION

Spontaneous rupture and upper gastrointestinal bleeding or haemoperitoneum should be considered a part of SPN clinical presentation.

A long-term follow-up should be performed in patients with ruptured SPN for neoplastic recurrence.

The relationship between the spontaneous rupture of SPN and the recurrence risk remains to be elucidated.

## CONFLICT OF INTEREST STATEMENT

The authors declare that there is no conflict of interest.

## FUNDING

None.
